# Interface engineering of CeO_2_ nanoparticle/Bi_2_WO_6_ nanosheet nanohybrids with oxygen vacancies for oxygen evolution reactions under alkaline conditions†

**DOI:** 10.1039/d2ra08273j

**Published:** 2023-03-16

**Authors:** Dukhyun Nam, Geunhyeong Lee, Jooheon Kim

**Affiliations:** a School of Chemical Engineering & Materials Science, Chung-Ang University 84 Heukseok-ro, Dongjak-gu Seoul Korea jooheonkim@cau.ac.kr; b Department of Advanced Materials Engineering, Chung-Ang University Anseong-si Gyeonggi-do 17546 Republic of Korea; c Department of Intelligent Energy and Industry, Graduate School, Chung-Ang University Seoul 06974 Republic of Korea

## Abstract

Because of the interactive combination synergy effect, hetero interface engineering is used way for advancing electrocatalytic activity and durability. In this study, we demonstrate that a CeO_2_/Bi_2_WO_6_ heterostructure is synthesized by a hydrothermal method. Electrochemical measurement results indicate that CeO_2_/Bi_2_WO_6_ displays not only more OER catalytic active sites with an overpotential of 390 mV and a Tafel slope of 117 mV dec^−1^ but also durability for 10 h (97.57%). Such outstanding characteristics are primarily attributed to (1) the considerable activities by CeO_2_ nanoparticles uniformly distributed on Bi_2_WO_6_ nanosheets and (2) the plentiful Bi–O–Ce and W–O–Ce species playing the role of strong couples between CeO_2_ nanoparticles and Bi_2_WO_6_ nanosheets and oxygen vacancy existence in CeO_2_ nanoparticles, which can improve the electrochemical active surface area (ECSA) and activity, and enhance the conductivity for OERs. This CeO_2_/Bi_2_WO_6_ consists of the heterojunction engineering that can open a modern method of thinking for high effective OER electrocatalysts.

## Abstract 2

The oxygen vacancies of catalysts enhance the electrocatalytic activity. CeO_2_/Bi_2_WO_6_ is synthesized *via* a hydrothermal reaction and NaBH_4_ reduction.

## Graphical abstract

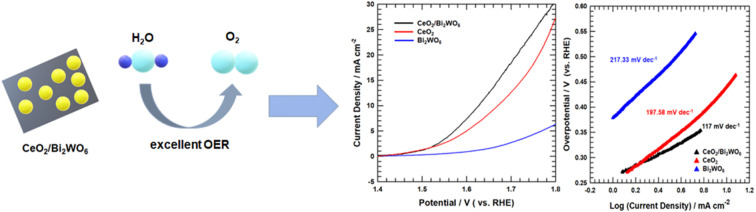

## 1. Introduction

The energy demands and increasing environmental problem lead to a lot of research efforts in studying exchangeable conversion system and energy storage.^1–6^ The oxygen evolution reaction (OER) is key to the progress of renewable energy devices such as water-splitting devices and metal–air batteries.^7–17^ At the anode, the even work of the OER depends on catalyst engineering owing to its essentially sluggish reaction kinetics and multielectron transfer paths.^18–22^ Generally, noble metal oxides such as IrO_2_ and RuO_2_ are well-known electrocatalysts for OERs.^23–27^ However, their high price, serious scarcity, and unsatisfied stability of electrocatalysts are greatly frustrating in that they are more widely applied to a variety of energy devices. Therefore, it is crucial to explore effective, low-cost, abundant, and robust OER catalysts on Earth.

One of the easiest members of the Aurivillius family, bismuth tungstate (Bi_2_WO_6_) has become an outstanding OER electrocatalyst because of its abundant, low cost, clean properties, and excellent chemical stability.^28–31^ In detail, two-dimensional Bi_2_WO_6_ nanosheets have a distinctive layer form and large specific surface area. These are useful to charge transfer, electrolyte penetration as well as active site exposure, regarded as a favorable catalyst support.^32,33^ Nonetheless, by the self-aggregating motion, Bi_2_WO_6_ nanosheets are simply aggregated to limit and decrease the electrochemically active region, indicating that the catalytic activity of OER is low.^30^ According to the surface structure, the adsorption actions of reaction region and charge distribution are crucial to the electrochemical catalytic action.^34^ Therefore, the interface engineering of heterostructures has been regarded as an effective strategy for optimizing the catalyst activities.^35–40^ The close connections between different active species in engineering interfaces optimize the powerful synergistic effect, rapid charge transfer rate and activation energy, and adsorption for intermediates, overcoming the shortcomings of single ingredient materials,^36–38^ whereas the heterointerfaces usually involve structural modification such as edges and dislocations as well as atomic defects including cation and anion vacancies, forming more active sites on the surface of the catalyst.^41^ To accomplish this aim, it is essential to choose appropriate introduced species that form the optimal electrocatalysts. Due to its chemical properties and unique electronic structure, CeO_2_ has been extensively studied as an effective supporter of the OER. The abundant oxygen vacancies of CeO_2_ and flexible conversion between Ce^3+^ and Ce^4+^ can enable several moving oxygen atoms to access active sites as an oxygen buffer for the effective absorption of oxygen species.^42–47^ Thus, we think that the hybridization of Bi_2_WO_6_ and CeO_2_ has to be a reasonable tactic to enhance the OER activity by the interface engineering.

In this work, we manufacture a modern sort of CeO_2_/Bi_2_WO_6_ heterostructure consisting of CeO_2_ nanoparticles on Bi_2_WO_6_ nanosheets by a hydrothermal method for OER electrocatalysts in alkaline media. The excellent electrocatalytic active site and durability come from the distinct heterostructure and combined interface synergistic effect with equally distributed CeO_2_ nanoparticles fixing Bi_2_WO_6_ nanosheets, which disclose more activity, have charge transfer rates, and show steady heterostructures. At the heterostructure, this approach *via* bonding the shape plan and electronic transformation fulfills advancement of catalysts, which supply direction for using activity encouraging and high effectiveness and stability OER electrocatalysts.

## 2. Experiment method

### 2.1. Synthesis of Bi_2_WO_6_ nanosheets

First, 0.05 g hexadecyltrimethylammonium bromide (CTAB) (0.1 mmol) was dispersed in 80 ml deionized water under stirring for 10 minutes. Then, 0.917 g Bi(NO_3_)_3_·5H_2_O was added to the obtained solution for 30 minutes. Finally, 0.33 g Na_2_WO_4_·2H_2_O was added to the solution and stirred for 30 minutes. Afterward, the as-obtained solution was transferred to a 100 ml Teflon-lined hydrothermal autoclave, which was then maintained at 120 °C for 24 hours. Finally, the precursor was washed several times with deionized water and dried at 50 °C overnight.

### 2.2. Synthesis of CeO_2_/Bi_2_WO_6_ nanohybrids

First, 1 mmol Ce(NH_4_)_2_(NO_3_)_6_ (0.5482 g) was added into 50 ml deionized water under a stirring process for 30 minutes. Then, 0.5 mmol Bi_2_WO_6_ (0.3488 g) was added to this solution and ultrasonicated until complete dissolution. After sonication, 10 ml of NaBH_4_ solution (0.05 M) was added to the solution. The product was washed several times with ethanol and deionized water and dried at 50 °C overnight. After drying overnight, the as-prepared sample was calcined at 420 °C for 2 hours.

### 2.3. Synthesis of CeO_2_ nanoparticles

The synthesis of CeO_2_ is similar to that of the CeO_2_/Bi_2_WO_6_ nanohybrid except for the additional treatment. To be more specific, although other experimental methods remain the same, only the second process of the CeO_2_/Bi_2_WO_6_ nanohybrid synthesis method was excluded.

## 3. Results and discussion

**Fig. 1:**
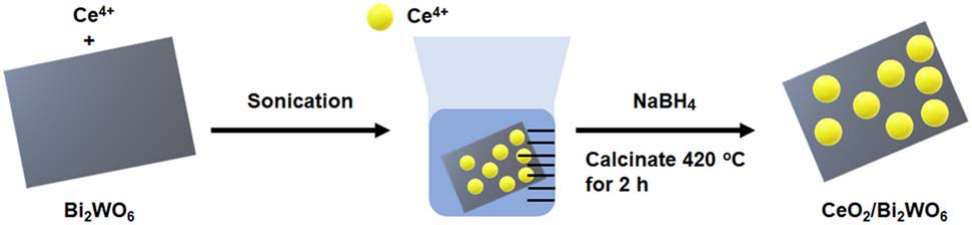
Schematic illustration of synthesis process of CeO_2_/Bi_2_WO_6_ nanohybrids.

Fig. 1 describes the process of formation of CeO_2_/Bi_2_WO_6_ nanohybrids *via* a hydrothermal reaction. The first process began with use of hydrothermal synthesis of Bi_2_WO_6_ nanosheets. The Br-ion CTAB bound on the Bi_2_WO_6_ surface can adsorb positively charged Ce^4+^ ions.^31,48^ Next, Ce^4+^ ions were easily reduced to CeO_2_ nanoparticles by forming nanohybrids of CeO_2_/Bi_2_WO_6_ using NaBH_4_ as a reducing agent accumulated on the Bi_2_WO_6_ nanosheet.^31^ During the experiment, Bi-ions and W-ions could be reduced by NaBH_4_ that obtains the advantages of Bi–O–Ce, W–O–Ce bond formation by substituting Br– to Ce–O–. The CeO_2_/Bi_2_WO_6_ nanohybrid was annealed at 420 °C in air, and thus, stable fixed CeO_2_ nanoparticles were bonded to the Bi_2_WO_6_ surface. The NaBH_4_ reduction was selected because it is easy to perform and inexpensive for the manufacture of vacancies. In addition, it generates many defects for exposing more reactive sites and increases the conductivity.^49^

### 3.1. Morphology and structure of CeO_2_/Bi_2_WO_6_

**Fig. 2:**
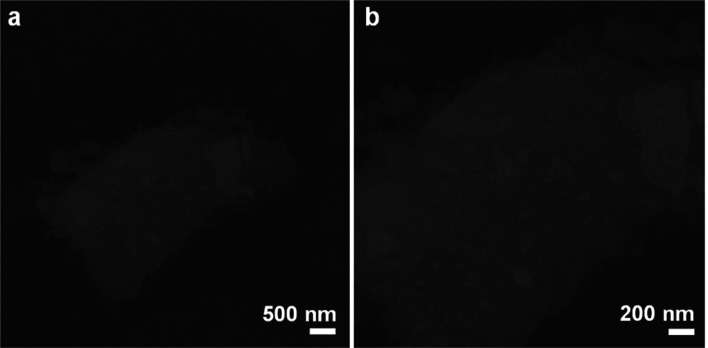
FE-SEM images at (a) low magnification and (b) high magnification of CeO_2_/Bi_2_WO_6_ nanohybrids.

The morphology and microstructure of the prepared samples were analyzed by FE-SEM, as shown in Fig. S1, S2† and 2. As shown in Fig. S1a and b (ESI†), the microstructure of the CeO_2_ sample was characterized by nanoparticles. The morphology of Bi_2_WO_6_ showed nanosheet features, as shown in Fig. S2a and b (ESI†). After addition of NaBH_4_ and Ce(NH_4_)_2_(NO_3_)_6_ and calcination at 420 °C for 2 hours, CeO_2_/Bi_2_WO_6_ could not change the structure of Bi_2_WO_6_ nanosheets (Fig. 2), which implies that the microstructure of Bi_2_WO_6_ could be maintained by the addition of NaBH_4_ and Ce(NH_4_)_2_(NO_3_)_6_ and calcination could keep the microstructure of Bi_2_WO_6_. In addition, the surface nanoparticles cannot be found on the CeO_2_/Bi_2_WO_6_ nanohybrid due to the low loading and uniform growth on the Bi_2_WO_6_ nanosheet of CeO_2_ nanoparticles.^28,29^ Meanwhile, the irregular nanoparticles on the surface could be distinguished from the surface of the Bi_2_WO_6_ nanosheets. This suggests that the CeO_2_ nanoparticles were successfully fixed and uniformly grown on the Bi_2_WO_6_ nanosheets. The distinctive heterostructure provided strong electron interaction and interfacial synergy between Bi_2_WO_6_ nanosheets and CeO_2_ nanoparticles, which is important for adjusting the electronic structure and exposing several active sites to increase the electrocatalytic activity and durability of electrocatalysts.^50,51^

**Fig. 3:**
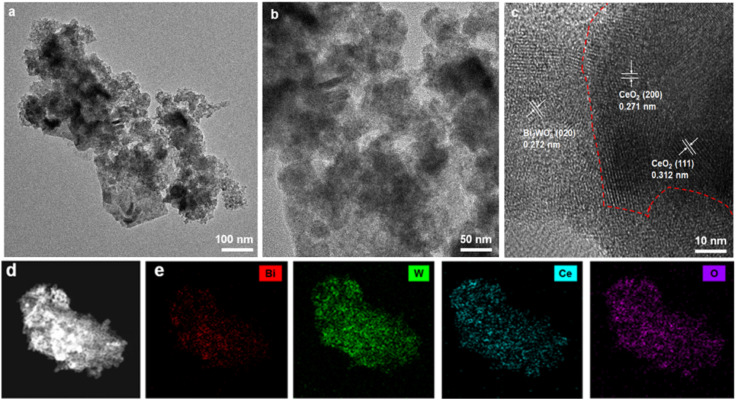
FE-TEM images at (a) low magnification and (b) high magnification of CeO_2_/Bi_2_WO_6_ nanohybrids. (c) HRTEM image of CeO_2_/Bi_2_WO_6_ nanohybrids. (d) Dark-field FE-TEM image of CeO_2_/Bi_2_WO_6_ nanohybrids. (e) EDS mapping images for Bi, W, Ce, and O elements distributed at CeO_2_/Bi_2_WO_6_ nanohybrids.

To further examine the structure of CeO_2_ nanoparticles on the surface of Bi_2_WO_6_ nanosheets, the crystal structure of CeO_2_, Bi_2_WO_6_, and CeO_2_/Bi_2_WO_6_ was investigated by FE-TEM analysis, as shown in Fig. S3, S4† and 3 As illustrated in Fig. S3a and b,† the FE-TEM images displayed CeO_2_ with nanoparticle structure, implying that the CeO_2_ nanoparticles were synthesized. The HRTEM image of CeO_2_ indicated that the *d*-spacing of the lattice fringes is 0.271 and 0.312 nm, corresponding to the (200) and (111) planes, respectively, as shown in Fig. S3c†.^52^ Meanwhile, the FE-TEM images represented Bi_2_WO_6_ with a sheet-like form, and the nanosheets can be seen in Fig. S4a and b,† showing that Bi_2_WO_6_ nanosheets were synthesized. As shown in Fig. S4c,† the HRTEM image shows that the *d*-space of lattice fringes is 0.272 nm, corresponding to the (020) plane of Bi_2_WO_6_.^53^ The FE-TEM images of CeO_2_/Bi_2_WO_6_ nanohybrids are displayed in Fig. 3a and b. The CeO_2_/Bi_2_WO_6_ sample was large and had nanosheet properties, and irregular CeO_2_ nanoparticles were dispersed on the Bi_2_WO_6_ nanosheets. In addition, it could be found that some nanoparticles were spread out on the Bi_2_WO_6_ nanosheets, confirming that the CeO_2_ nanoparticles were grown on the Bi_2_WO_6_ nanosheets, which is consistent with the FE-SEM results.^54^Fig. 3c shows the HRTEM image of the CeO_2_/Bi_2_WO_6_ nanohybrid catalyst, and the lattice edges of CeO_2_ nanoparticles and Bi_2_WO_6_ nanosheets might be surely differentiated, and the lattice edges of 0.271 nm, 0.312 nm, and 0.272 nm corresponded to the (200) and (101) planes of CeO_2_ and the (020) plane of Bi_2_WO_6_, respectively. Finally, to investigate the elemental composition of CeO_2_/Bi_2_WO_6_ nanohybrids catalyst, the energy dispersive X-ray spectrometry (EDS) was perfected in Fig. 3e. The four elements of Ce, Bi, W, and O were uniformly distributed over the whole CeO_2_/Bi_2_WO_6_ nanohybrid catalyst, which suggested that the CeO_2_ nanoparticles combined with the surface of Bi_2_WO_6_ nanosheets, confirming that the CeO_2_ nanoparticle/Bi_2_WO_6_ nanosheet heterostructure was successfully synthesized.

**Fig. 4:**
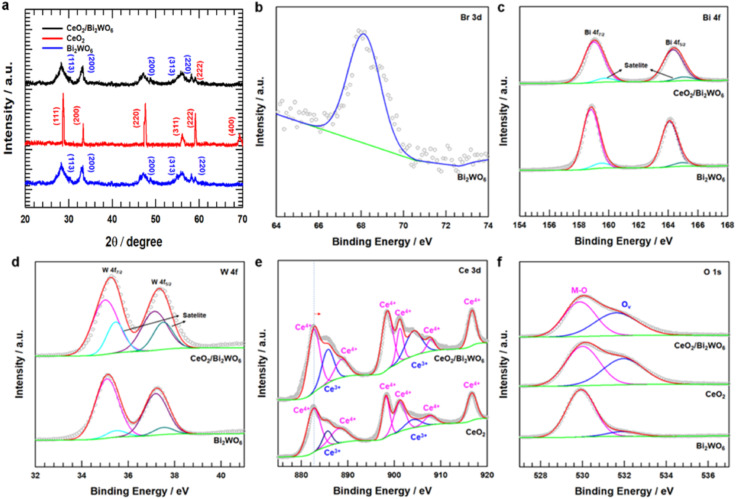
(a) XRD pattern of CeO_2_/Bi_2_WO_6_, and CeO_2_/Bi_2_WO_6_. (b) XPS Br 3d deconvolution spectrum of Bi_2_WO_6_. (c) XPS Bi 4f deconvolution spectrum of Bi_2_WO_6_ and CeO_2_/Bi_2_WO_6_. (d) XPS W 4f deconvolution spectrum of Bi_2_WO_6_ and CeO_2_/Bi_2_WO_6_. (e) XPS Ce 3d deconvolution spectrum of CeO_2_ and CeO_2_/Bi_2_WO_6_. (f) XPS O 1s deconvolution spectrum of CeO_2_, Bi_2_WO_6_ and CeO_2_/Bi_2_WO_6_.

To confirm the crystal structure and phase composition of CeO_2_/Bi_2_WO_6_, CeO_2_, and Bi_2_WO_6_ catalysts, we conducted X-ray diffraction (XRD), as shown in Fig. 4a. The peaks at 28.7°, 33.3°, 47.6°, 56.5°, 59.3°, and 69.5° corresponded to the (111), (200), (220), (311), (222), and (400) planes of CeO_2_, respectively. These results were consistent with the CeO_2_ crystal structure (JCPDS No. 81-0792).^55^ Similarly, the diffraction peaks of CeO_2_/Bi_2_WO_6_ and Bi_2_WO_6_ matched JCPDS No. 73-2020 of Bi_2_WO_6_.^56^ In addition, no diffraction peaks were studied from other materials. This might be the surface of the Bi_2_WO_6_ nanosheets of the CeO_2_ nanoparticles due to low loading and even growth.^29–31^

To identify the chemical valence states and surface elemental contents, the X-ray photoelectron (XPS) spectra recorded for CeO_2_, Bi_2_WO_6_, and CeO_2_/Bi_2_WO_6_ are shown in Fig. S5† and 4b–f. As shown in Fig. S5,† the XPS survey spectrum indicated the existence of Ce, Bi, W, and O elements, in accordance with the above-mentioned XRD result (Fig. 4a). Fig. 4b–f displays the high-resolution spectra of Br 3d, Bi 4f, W 4f, Ce 3d and O 1s, respectively. In the case of pure Bi_2_WO_6_, the binding energies of the Br 3d peak were determined to be 68.6 eV, as shown in Fig. 4b, confirming that the Br ions of CTAB were bound to the surface Bi and W atoms of Bi_2_WO_6_.^53^ As shown in Fig. 4c, Bi_2_WO_6_ and CeO_2_/Bi_2_WO_6_ could be divided into two Bi 4f peaks. The properties of Bi 4f_5/2_ and Bi 4f_7/2_ were two peaks at 164.3 and 159.2 eV that matched Bi^3+^ ions of Bi_2_WO_6_.^57^ The shoulder peaks Bi 4f_5/2_ and Bi 4f_7/2_, corresponding to 165.6 and 160.6 eV, appeared at a higher binding energy. The peaks of Bi, represented at a higher energy, meant that the Bi atoms had higher electrical positivity in binding with the surface Br atoms.^53,57^ Similarly, for the high-revolution XPS W 4f spectrum (Fig. 4d), 4f_7/2_ and 4f_5/2_ electron orbits of W^6+^ corresponded to two feature peaks at 35.2 eV and 37.3 eV, respectively. In addition, the orbits of W 4f_7/2_ and W 4f_5/2_ belonged to the satellite peaks at 35.6 eV and 37.6 eV, respectively. Compared to Bi_2_WO_6_, the binding energy of CeO_2_/Bi_2_WO_6_ was moved slightly to the negative parts, confirming that the electropositive W appearing on the Bi_2_WO_6_ nanosheets was increasingly higher.^28,53^ The high-resolution XPS Ce 3d spectrum for CeO_2_/Bi_2_WO_6_ was composed with the peaks compared to CeO_2_ (Fig. 4e). The Ce 3d spectrum of CeO_2_/Bi_2_WO_6_ and CeO_2_ samples could be separated into eight peaks, two peaks were assigned to Ce^3+^ at 885.7 and 904.2 eV, and six peaks were assigned to Ce^4+^ at 882.7, 888.7, 898.5, 901.2, 907.9, and 916.7 eV for CeO_2_/Bi_2_WO_6_.^58^ According to the Ce 3d spectrum analysis, Ce^3+^ and Ce^4+^ were present in CeO_2_ and CeO_2_/Bi_2_WO_6_. For the Ce 3d spectrum, it might be observed that CeO_2_ and CeO_2_/Bi_2_WO_6_ were plentiful in Ce^3+^ species, which showed the formation of oxygen vacancies in these two samples.^58^ Besides, the binding energy of the Ce 3d spectrum in CeO_2_/Bi_2_WO_6_ had a clear positive change compared to CeO_2_. The suitable electron structure of CeO_2_/Bi_2_WO_6_ could help to enhance the catalyst's OER performance by inducing charge redistribution at the interface.^59,60^Fig. 4f shows the two peaks for the O 1s spectrum. The O 1s peak at 530.2 eV was attributed to the oxygen atom bonded to the metal, and the center position at 532.1 eV was ascribed to the oxygen atom in the surrounding area of oxygen vacancies.^61^ However, according to the feature peak, the peak area at 532.1 eV varied greatly, which displayed that the CeO_2_/Bi_2_WO_6_ nanohybrids had much more oxygen vacancies. Interestingly, as shown in Table S1,† the CeO_2_/Bi_2_WO_6_ nanohybrids (46.8%) is higher than that of CeO_2_ nanoparticles (44.6%) and Bi_2_WO_6_ nanosheets (9.7%). These results indicated that the CeO_2_/Bi_2_WO_6_ nanohybrids had enough oxygen vacancies. As a result, the CeO_2_ nanoparticles abundant in evenly grown oxygen vacancies on Bi_2_WO_6_ nanosheets were successfully synthesized.

### 3.2. Oxygen electrochemical performance of electrocatalysts

**Fig. 5:**
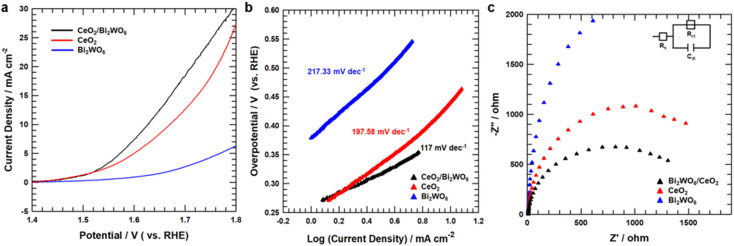
(a) OER LSV curves for CeO_2_, Bi_2_WO_6_, and CeO_2_/Bi_2_WO_6_ in a N_2_-saturated 1.0 M KOH electrolyte. (b) Tafel plots for CeO_2_, Bi_2_WO_6_, and CeO_2_/Bi_2_WO_6_. (c) Nyquist plots for CeO_2_, Bi_2_WO_6_, and CeO_2_/Bi_2_WO_6_ recorded at 1.65 V.

To study the OER catalytic active sites of all samples, we studied the electrochemical characteristics of CeO_2_/Bi_2_WO_6_, CeO_2_, and Bi_2_WO_6_ for OERs in alkaline solutions (pH = 14) using a rotating disk electrode (RDE) (see Detail Methods in the ESI†). As shown in Fig. 5a, the linear sweep voltammetry (LSV) curves showed that CeO_2_/Bi_2_WO_6_ indicated a smaller overpotential of 390 mV, slightly larger than that of CeO_2_ (440 mV) and Bi_2_WO_6_. Besides, to evidence the outstanding OER kinetics of the samples, their Tafel slope were calculated by LSV. As shown in Fig. 5b, CeO_2_/Bi_2_WO_6_ showed a lower Tafel slope (117 mV dec^−1^) than that of CeO_2_ (197.58 mV dec^−1^) and Bi_2_WO_6_ (217.33 mV dec^−1^), and thus CeO_2_/Bi_2_WO_6_ had the fastest kinetic process.^62,63^ Compared with previous studies, the CeO_2_/Bi_2_WO_6_ heterostructure was one of the most efficient Bi_2_WO_6_-based catalysts (Table S2†). The smallest Tafel slope of CeO_2_/Bi_2_WO_6_ suggested the most favorable OER kinetics, indicating that CeO_2_/Bi_2_WO_6_ possessed an outstanding OER catalytic kinetics. To investigate the OER kinetics of CeO_2_/Bi_2_WO_6_, CeO_2_, and Bi_2_WO_6_, electrochemical impedance spectroscopy (EIS) was conducted, as shown in Fig. 5c. The CeO_2_/Bi_2_WO_6_ nanohybrids had the lowest charge resistance (*R*_ct_) than other samples at the interface between the electrolyte and the catalyst. Since *R*_ct_ represented the rate of charge transfer in OERs,^64^ the smallest *R*_ct_ value of the CeO_2_/Bi_2_WO_6_ nanohybrid showed the high-speed electron transportation ability of the CeO_2_/Bi_2_WO_6_ nanohybrid during the OER process due to the CeO_2_ nanoparticles plentiful in oxygen vacancies evenly grown on Bi_2_WO_6_ nanosheets.^28^

**Fig. 6:**
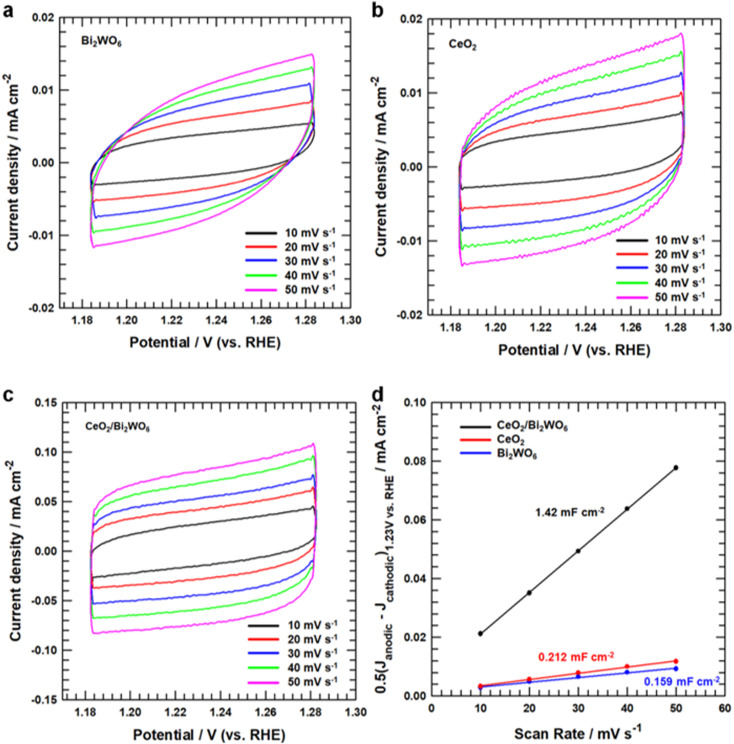
CV curves (a) CeO_2_, (b) Bi_2_WO_6_, and (c) CeO_2_/Bi_2_WO_6_ in a non-faradaic current region (1.18–1.28 V *vs.* RHE) at different scan rates of 10, 20, 30, 40, and 50 mV s^−1^. (d) Linear fitting of the capacitive currents *versus* CV scan rates of CeO_2_, Bi_2_WO_6_, and CeO_2_/Bi_2_WO_6_.

To establish why CeO_2_/Bi_2_WO_6_ had better OER activity than that of other samples, we measured double-layer capacitance (*C*_dl_) to judge their electrochemically active surface area (ECSA). The ECSA of CeO_2_/Bi_2_WO_6_, CeO_2_, and Bi_2_WO_6_ was revealed by a cyclic voltammetry (CV) method.^65–67^Fig. 6a–c display the CV curves at different scan rates (10–50 mV s^−1^) for CeO_2_/Bi_2_WO_6_, CeO_2_, and Bi_2_WO_6_ alkaline solutions, respectively. As the scan speed increased, the current densities of CeO_2_/Bi_2_WO_6_, CeO_2_, and Bi_2_WO_6_ increased accordingly, indicating that the active sites and charge transport capability of CeO_2_/Bi_2_WO_6_, CeO_2_, and Bi_2_WO_6_ increased significantly. In addition, it displayed that CeO_2_/Bi_2_WO_6_ showed the highest capacitive current compared with CeO_2_ and Bi_2_WO_6_. The *C*_dl_ and ECSA can be calculated as “0.5(*J*_anodic_–*J*_cathodic_)_1.23 V *vs.* RHE_ (mA cm^−2^)/scan rate (mV s^−1^)”, as shown in Fig. 6d, and the *C*_dl_ of CeO_2_/Bi_2_WO_6_ (1.42 mF cm^−2^) is remarkably higher than that of CeO_2_ (0.212 mF cm^−2^) and Bi_2_WO_6_ (0.159 mF cm^−2^). As a result, the significant activities of *C*_dl_ and ECSA increased, which might be due to the high oxygen vacancy concentration of the CeO_2_/Bi_2_WO_6_ heterostructure, and CeO_2_ nanoparticles equally grown on Bi_2_WO_6_, which essentially improved the electrocatalytic activity.

**Fig. 7:**
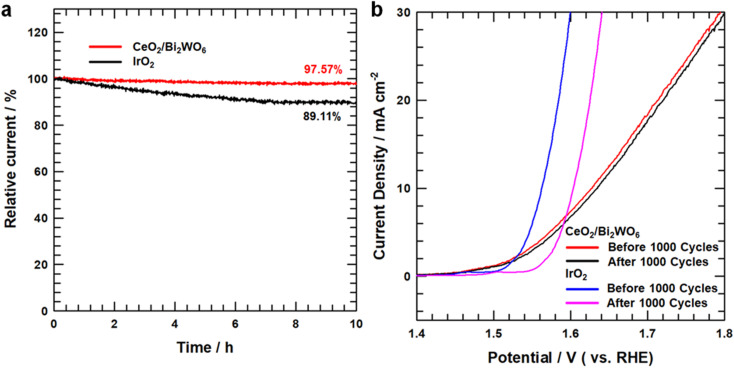
(a) OER chronoamperometry test of CeO_2_/Bi_2_WO_6_ and IrO_2_. (b) OER LSV curves for before and after 1000 cycles CeO_2_/Bi_2_WO_6_ and IrO_2_.

The electrocatalytic stability of the CeO_2_/Bi_2_WO_6_ nanohybrids and IrO_2_ was tested by chronoamperometry measurements, as shown in Fig. 7a, and the current density of CeO_2_/Bi_2_WO_6_ indicated the unseen modification with respect to the initial value at a retention rate of up to 97.57% after 10 hours of the OER process and showed outstanding stability in an aqueous alkaline medium. In IrO_2_, the current retention rate is below 89.11%. Besides, the durability of CeO_2_/Bi_2_WO_6_ was performed by the LSV curves before and after 1000 cycles of the CV curves. As shown in Fig. 7b, the CeO_2_/Bi_2_WO_6_ electrocatalyst showed a negligible decrease in current density, suggesting the good durability of CeO_2_/Bi_2_WO_6_ in alkaline solutions, while IrO_2_ shows a significant decrease after 1000 cycles. Because of the synergistic effect of highly stable heterojunctions, the Bi_2_WO_6_ nanosheets not only guarantee rich active sites, but also ensure a variety of paths for the fast and efficient movement of electrolytes and gases. Meanwhile, the reasonably fixed CeO_2_ nanoparticles increase the electrocatalytic activity and enhance the electrical contact with the electrolyte.^68,69^ The above-mentioned electrochemical results confirmed the presence of more active sites, and more efficient and faster electron transport capability in CeO_2_/Bi_2_WO_6_ than those in samples of CeO_2_ and Bi_2_WO_6_, confirming that the CeO_2_/Bi_2_WO_6_ heterostructure catalyst had fine catalytic activity and maintained the excellent stability in an alkaline environment. Therefore, the CeO_2_/Bi_2_WO_6_ heterostructure catalyst is a reasonable strategy to optimize the OER active sites and durability of Bi_2_WO_6_-based catalysts.

## 4. Conclusion

In summary, we have developed a simple strategy to synthesize CeO_2_/Bi_2_WO_6_ nanohybrids with more OER active sites and high durability under alkaline conditions. The characterization and electrochemical measurement results indicated that the CeO_2_/Bi_2_WO_6_ heterostructure electrocatalyst displayed not only more OER catalytic active sites with a smaller overpotential of 390 mV and a lower Tafel slope of 117 mV dec^−1^ but also durability for 12 h. The distinct heterointerface generates hard bonded electronic effects and the interfacial synergistic effect, making the CeO_2_ nanoparticles uniformly anchored onto Bi_2_WO_6_ for the atoms to expose more active sites, which provided CeO_2_/Bi_2_WO_6_ with electrocatalytic active sites for OERs. Meanwhile, the hard coupled and interfacial synergistic effect really endows the heterojunction structure with good stability for practical application. This CeO_2_/Bi_2_WO_6_ heterostructure catalyst has been developed *via* shape design.

## Conflicts of interest

There are no conflicts to declare.

## Supplementary Material

RA-013-D2RA08273J-s001
