# Supplementary material for: Interface engineering of CeO2 nanoparticle/Bi2WO6 nanosheet nanohybrids with oxygen vacancies for oxygen evolution reactions under alkaline conditions
Source: RSC Adv. 2023 Mar 16;13(13):8873–81. doi: 10.1039/d2ra08273j (PMC10018795; doi:10.1039/d2ra08273j)
Supplement: RA-013-D2RA08273J-s001 [file RA-013-D2RA08273J-s001.pdf]

Electronic Supplementary Material (ESI) for RSC Advances.
This journal is © The Royal Society of Chemistry 2023

Supporting Information

Interface engineering of CeO_2 nanoparticles/Bi_2WO_6 nanosheets nanohybrid with oxygen vacancy for Oxygen Evolution Reaction in alkaline conditions

Dukhyun Nam[a], Geunhyeong Lee[a], Jooheon Kim[a,b,c] *

[a] School of Chemical Engineering & Materials Science, Chung-Ang University, 84 Heukseok-ro, Dongjak-gu, Seoul, Korea

[b] Department of Advanced Materials Engineering, Chung-Ang University, Anseong-si, Gyeonggi-do 17546, Republic of Korea

[c] Department of Intelligent Energy and Industry, Graduate School, Chung-Ang University, Seoul 06974, Republic of Korea

*Corresponding author

Prof. Jooheon Kim: jooheonkim@cau.ac.kr

Detail methods

Chracterization methods.

Images of the nanoparticles morphology were acquired by field emission scanning electron microscope (FE-SEM, SIGMA 300, Carl Zeiss, Korea). Then, images of the nanoparticles morphology and energy dispersive X-ray (EDX) mapping data were acquired by field emission transmission electron microscopy (FE-TEM, JEM-F200, JEOL, Korea). The amorphous phase of obtained catalysts was confirmed by X-ray diffraction (XRD, Bruker-AXS, country) using a Cu Kα radiation source at 40 kV (λ = 0.154 nm, scan rate = 1.2°/min, step size = 0.02° and diffraction range = $10^{o} \leq 2\theta \leq 80^{o}$). X-ray photoelectron spectroscopy (XPS, K-alpha, ThermoFisher Scientific, country) analysis was used to explore the electrochemical bond structure of the catalysts and to examine the effects of interaction between atoms.

Electrochemical activity measurements

Electrochemical performance of all samples was evaluated using three-electrode configuration with a potentiometry (CH Instruments, CHI 600E, USA) and a rotating-disk electrode (RDE). Before measurements, the glassy carbon electrode (GCE, diameter: 3.0 mm) was polished using 1 μm polishing diamond and 0.05 μm polishing alumina on the polishing pad, and then rinsed with DI water. The catalytic ink was prepared by mixing synthesized catalysts (5.0 mg), DI water (700 μL), isopropanol (200 μL) and Nafion solution (100 μL, 5 %, Alfa-Aesar). 8 μL of catalytic ink were dropped onto the GCE surface and left to dry for 1 h under 50 °C. Electrochemical properties of the catalysts were investigated using platinum wire (counter electrode), a GCE (working electrode) and an Ag/AgCl electrode (reference electrode). All electrochemical measurements were conducted in 1 M alkaline solution (KOH) with N_2 gas purged for at least 30 minutes, maintaining N_2 saturation during the measurements. All electrochemical data were calibrated with respect to the reversible hydrogen electrode (RHE), following the previously reported conversion equation ($E_{RHE} = E_{Ag/AgCl} + 0.0592$ pH

+ $E_{Ag/AgCl}^0$ at 1 M KOH media, where, $E_{Ag/AgCl}^0$ (in 1 M KCl) = +0.235 V, pH = 14 for 1 M KOH), and also corrected by iR-compensation, to remove the ohmic potential loss, according to equation (1):

$$E_c = E_m - I_m \times R_s \quad (1)$$

Where, E_c, E_m, I_m and R_s correspond to corrected voltage, measured voltage, measured current and electrolyte resistance, respectively. Electrochemical impedance spectroscopy (EIS) was recorded from 100 kHz to 0.1 Hz at 1.7 V (vs. RHE) with amplitude of 10 mV.

The overpotential was examined by LSV, using 1600 rpm at a 5 mV/s scan rate, and a voltage range of 0.2-1.0 V in Ag/AgCl. The Tafel slope was obtained from the LSV curve by applying equation (2):

$$\eta = a + b \times \log|J| \quad (2)$$

Where, b, J, and η are the Tafel slope, current density and overpotential, respectively.

In order to evaluate the electrochemically active surface area (ECSA), cyclic voltammetry (CV) curve was obtained using a voltage range of 1.12 V to 1.22 V in RHE (no Faradaic process voltage range) with various scan rates, and the linear relationship between scan rates and current densities was estimated. As a result, we obtained the double-layer capacitance (C_{dl}) using equation (3).

$$C_{dl} = a \times \frac{|ja - jc|}{2v} \quad (3)$$

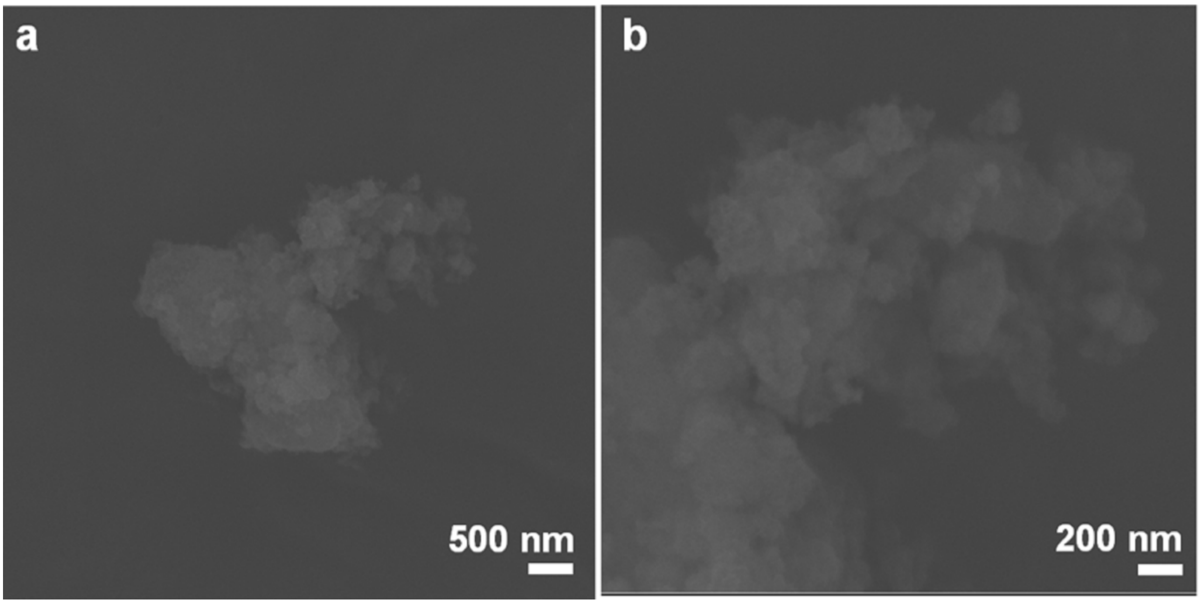

Fig. S1. FE-SEM images at (a) low magnification and (b) high magnification of CeO_2 nanoparticles.

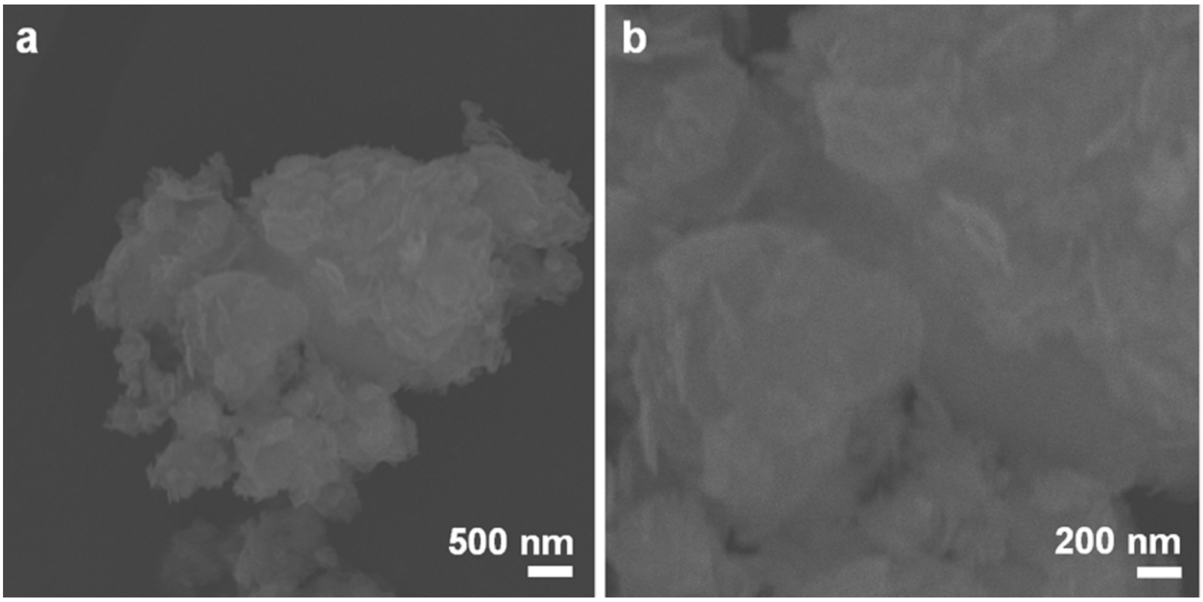

Fig. S2. FE-SEM images at (a) low magnification and (b) high magnification of Bi_2WO_6 nanosheets.

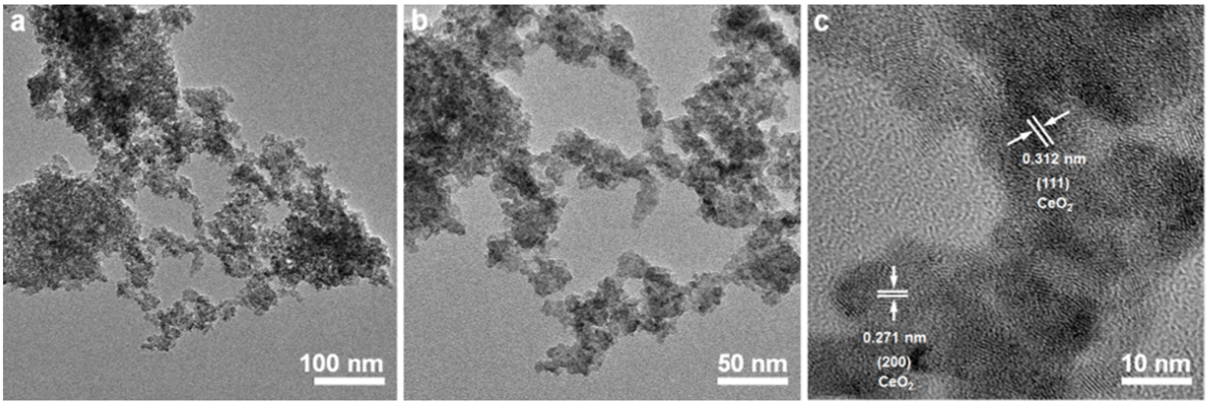

Fig. S3. FE-TEM images at (a) low magnification and (b) high magnification of CeO_2 nanoparticles. (c) HRTEM image of CeO_2 nanoparticles.

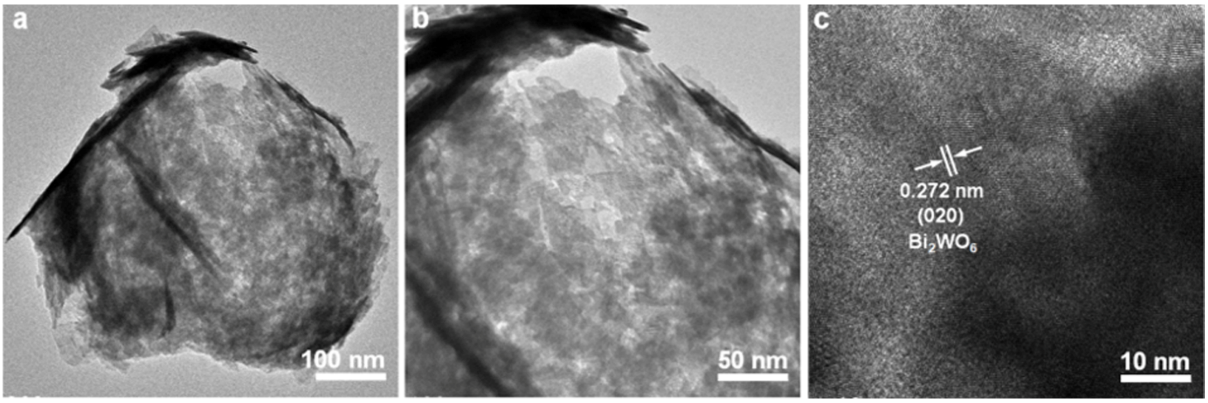

Fig. S4. FE-TEM images at (a) low magnification and (b) high magnification of Bi_2WO_6 nanosheets. (c) HRTEM image of Bi_2WO_6 nanosheets.

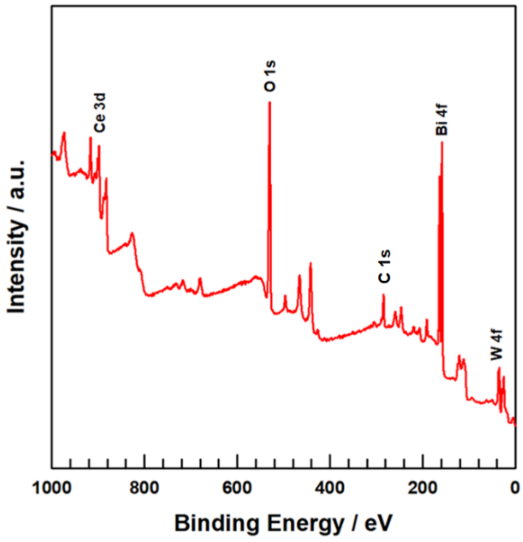

Fig. S5. XPS survey spectra for CeO_2/Bi_2WO_6.

	Oxygen Vacancies
CeO_2/Bi_2WO_6	46.8 %
CeO_2	44.6 %
Bi_2WO_6	9.7 %

Table S1. the oxygen vacancies amounts of CeO_2/Bi_2WO_6, CeO_2 and Bi_2WO_6.

	OER E(@10mA cm-2) /V (vs. RHE)	OER Tafel slope / mV dec-1	Reference
Bi_2WO_6 CNPs	1.77	-	[S1]
CoO_x/Bi_2WO_6-2	1.72	114	[S2]
NiS/Bi_2WO_6-4	1.757	238	[S3]
$NiFeO_x/Bi_2WO_6$	1.73	125	[S4]
CeO_2/Bi_2WO_6	1.62	117	This work

Table S2. The OER performance of CeO_2/Bi_2WO_6 in this work and some recently reported alkaline electrolyte.

	R_s (Ω)	R_{ct} (Ω)
CeO_2/Bi_2WO_6	11.93	1302.56
CeO_2	13.29	1498.23
Bi_2WO_6	13.94	4034.65

Table S3. Comparison of solution resistance (R_s) and charge transfer resistance (R_{ct}) for OER.

Reference

[S1] Z.-P. Nie, D.-K. Ma, G.-Y. Fang, W. Chen, S.-M. Huang, Concave Bi2WO6 nanoplates with oxygen vacancies achieving enhanced electrocatalytic oxygen evolution in near-neutral water, J. Mater. Chem. A, 4 (2016) 2438-2444.

[S2] J. Li, Z. Liang, Q. Song, X. Xu, Strong-coupled CoOx nanoparticles/Bi2WO6 nanosheets hybrid as electrocatalyst for water oxidation under alkaline conditions, Mater. Res. Bull. 113 (2019) 152-160.

[S3] J. Li, X. Xu, B. Zhang, W. Hou, S. Lv, Y. Shi, Controlled synthesis and fine-tuned interface of NiS nanoparticles/Bi2WO6 nanosheets heterogeneous as electrocatalyst for oxygen evolution reaction, Appl. Surf. Sci., 526 (2020) 146718.

[S4] J. Li, Z. Liang, Q. Song, X. Xu, NiFeOx nanosheets tight-coupled with Bi2WO6 nanosheets to improve the electrocatalyst for oxygen evolution reaction, Appl. Surf. Sci., 478 (2019) 969-980.
